# Epidemiology of Visceral Leishmaniasis in Georgia

**DOI:** 10.1371/journal.pntd.0002725

**Published:** 2014-03-06

**Authors:** Giorgi Babuadze, Jorge Alvar, Daniel Argaw, Harry P. de Koning, Merab Iosava, Merab Kekelidze, Nikoloz Tsertsvadze, David Tsereteli, Giorgi Chakhunashvili, Tamar Mamatsashvili, Nino Beria, Irine Kalandadze, Mikhail Ejov, Paata Imnadze

**Affiliations:** 1 National Center for Disease Control and Public Health of Georgia, Tbilisi, Georgia; 2 Ilia State University, Tbilisi, Georgia; 3 WHO/NTD/Leishmaniasis Program, Geneva, Switzerland; 4 World Health Organization/NTD, Geneva, Switzerland; 5 Institute of Infection, Immunity and Inflammation, University of Glasgow, Glasgow, United Kingdom; 6 World Health Organization, Regional Office for Europe, Copenhagen, Denmark; Hebrew University-Hadassah Medical School, Israel

## Abstract

This study investigated the transmission and prevalence of *Leishmania* parasite infection of humans in two foci of Visceral Leishmaniasis (VL) in Georgia, the well known focus in Tbilisi in the East, and in Kutaisi, a new focus in the West of the country. The seroprevalence of canine leishmaniasis was investigated in order to understand the zoonotic transmission. Blood samples of 1575 dogs (stray and pet) and 77 wild canids were tested for VL by Kalazar Detect rK39 rapid diagnostic tests. Three districts were investigated in Tbilisi and one in Kutaisi. The highest proportions of seropositive pet dogs were present in District #2 (28.1%, 82/292) and District #1 (26.9%, 24/89) in Tbilisi, compared to 17.3% (26/150) of pet dogs in Kutaisi. The percentage of seropositive stray dogs was also twice as high in Tbilisi (16.1%, n = 670) than in Kutaisi (8%, n = 50); only 2/58 wild animals screened were seropositive (2. 6%). A total of 873 *Phlebotomine* sand flies were collected, with 5 different species identified in Tbilisi and 3 species in Kutaisi; 2.3% of the females were positive for *Leishmania* parasites. The Leishmanin Skin Test (LST) was performed on 981 human subjects in VL foci in urban areas in Tbilisi and Kutaisi. A particularly high prevalence of LST positives was observed in Tbilisi District #1 (22.2%, 37.5% and 19.5% for ages 5–9, 15–24 and 25–59, respectively); lower prevalence was observed in Kutaisi (0%, 3.2% and 5.2%, respectively; P<0.05). This study shows that Tbilisi is an active focus for leishmaniasis and that the infection prevalence is very high in dogs and in humans. Although exposure is as yet not as high in Kutaisi, this is a new VL focus. The overall situation in the country is alarming and new control measures are urgently needed.

## Author Summary

Leishmaniasis is a disease complex of various clinical manifestations caused by infection with protozoan parasites (*Leishmania* spp). It is transmitted through the bite of infected sand flies (*Phlebotomus* or *Lutzomyia* spp) and dogs are the main reservoir host for the *Leishmania infantum* species described previously in Georgia. It is prevalent in many tropical and subtropical regions of the world. In Georgia, visceral leishmaniasis has been known to occur in the East and in the capital, Tbilisi, but to date no investigation of vectors, and of prevalence in humans and in canines, has been conducted. Here, we report 5 different species of sand fly in Tbilisi and 3 in the West-Georgian city of Kutaisi, with infected vectors found in both places. In some districts of Tbilisi more than a quarter of pet dogs were seropositive for *Leishmania* parasites; prevalence in stray dogs was somewhat lower. Even in Kutaisi, where no leishmaniasis has previously been reported, 17.3% of pet dogs tested positive. This was reflected in high prevalence of infection in humans in the capital (14.5% overall), compared to 7.3% in Kutaisi. We conclude that the infection rate with visceral leishmaniasis in Georgia is alarmingly high and that its transmission has significantly spread west-wards in recent times.

## Introduction

**Figure 1:**
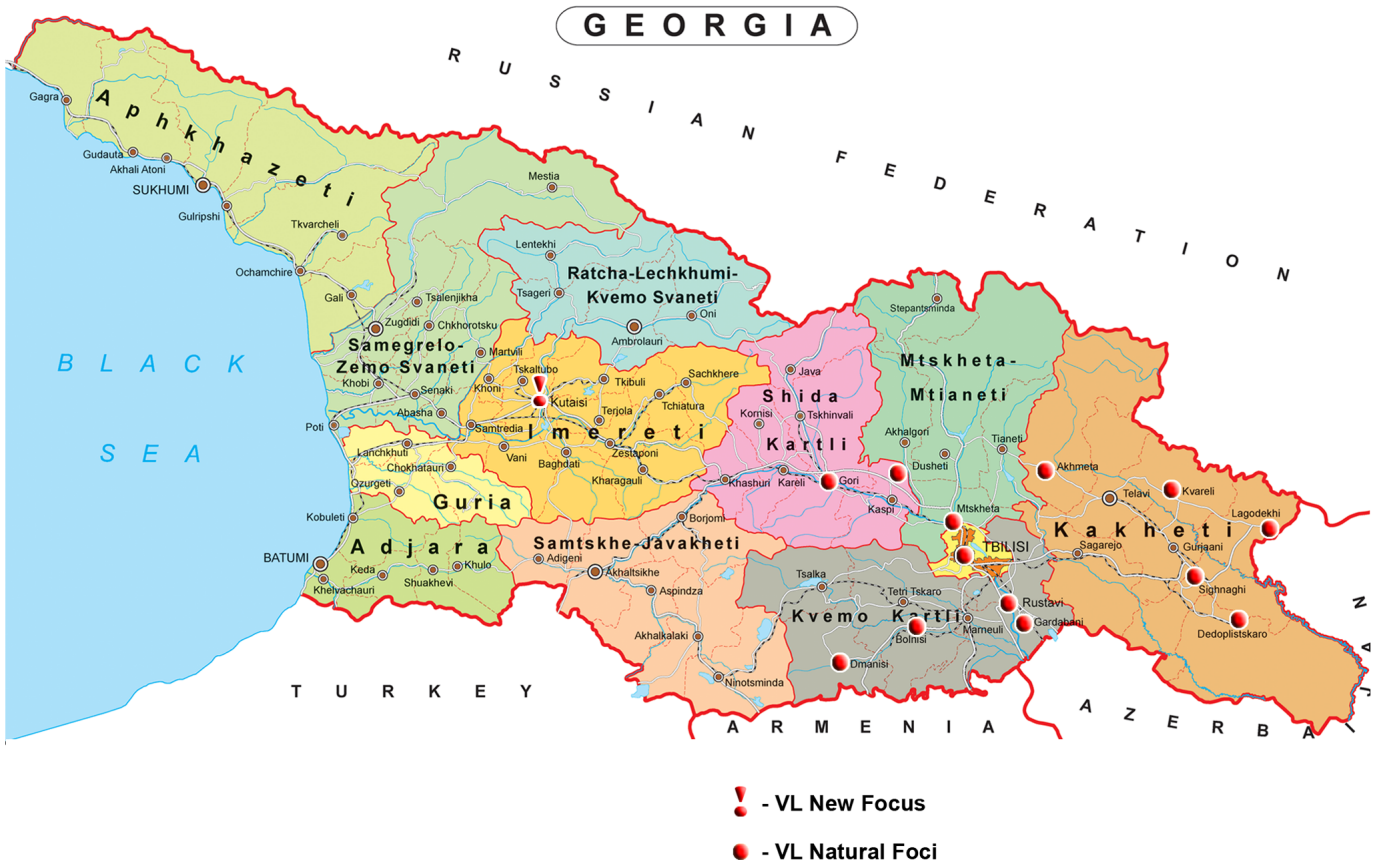
Map of Georgia with known VL foci indicated.

In recent years reports have emerged of increased leishmaniasis transmission in Europe [1], drug resistant leishmaniasis has spread further [2], and the spread of HIV/leishmaniasis co-infection is a trend of particular concern [3], [4].Visceral Leishmaniasis (VL) is mainly caused by two species of parasites, the anthroponotic *L. donovani* and the zoonotic *L. infantum*, for which a variety of canids serve as the animal reservoir. Most infections are asymptomatic, although longitudinal follow-up has shown that some infected individuals eventually predispose to clinical disease. Malnutrition and immune suppression, notably HIV infection, predispose to clinical disease, and children are especially affected [5]. Zoonotic VL occurs in many former Soviet Union countries and presents one of the most serious public health concerns in Georgia [5], [6]. Several natural VL foci have been identified in the country where various species of *Phlebotomus* and canine reservoirs facilitate the transmission (see Figure 1) [7].

The first four cases of VL in Georgia were described in 1913, which to the best of our knowledge was the first report about this disease in the entire Caucasus region. The first well-described outbreaks (540 cases) of VL were reported in eastern Georgia in 1954 [6], [8]. All cases were registered in 6 cities and 164 villages, mainly in the east of the country, in the Shida Kartly and Kakheti regions (see Figure 1).

Malaria control efforts in Eastern Georgia in the sixties included massive spraying campaigns with the insecticide dichlorodiphenyltrichloroethane (DDT) [8], which is believed to have also caused a significant reduction in the sand fly population, as during the next 40 years, until the 1990s, only sporadic VL cases were registered, and only in the extreme eastern part of the country. Since 2005, 19 VL fatal cases have been registered in Georgia, usually the result of late diagnosis and/or misdiagnosis. VL traditionally affects children in the 1–5 years age group. However in the last 5 years a relatively high number of adults have been among the reported VL cases, indicating that the disease may be re-emerging as an epidemic instead of the previously low endemic situation [official disease records, National Centre for Disease Control (NCDC), Tbilisi, Georgia].

The first VL case in Tbilisi was registered in 1990 [7]. Two cases of *Leishmania*/HIV co-infection were diagnosed in 2008; for both cases the outcome was fatal [official disease records, NCDC 2008]. Cutaneous leishmaniasis (CL) is less frequent: 125 CL cases were registered in the period 1928–1964, of which 110 (88.0%) occurred in Tbilisi and villages situated in the western part of the Mtkvari river valley –Shida Kartli region, 56 km west from Tbilisi (see Figure 1). After a long interval without registered cases, new cases of CL started to appear, and mandatory registration of CL started in 2001. Between 2001 and 2007, 1–5 cases of CL were reported in most years, which increased to 12 cases in 2008–2009, 8 of them in Tbilisi [official disease records, NCDC]. Both CL and VL are underreported, due to their relatively recent re-emergence, a consequent lack of awareness in the population, and the lack of a leishmaniasis training program for medical doctors. In Kutaisi the first VL cases were only registered as recently as 2007 (3 cases) [official disease records, NCDC 2007] in which year also the first sand flies of the *Phlebotomus* genus were identified in this area [G. Babuadze unpublished study].

During the last two decades, the annual number of clinical VL cases has remained persistently high, and varies between 122 and 189. In the period 1995–2010, 1919 VL cases were registered, including 1052 from Tbilisi [official disease records, NCDC]. Several natural VL foci have been identified, where several species of *Leishmania* vectors exist and canine reservoirs facilitate the transmission of this disease [7].

**Figure 2:**
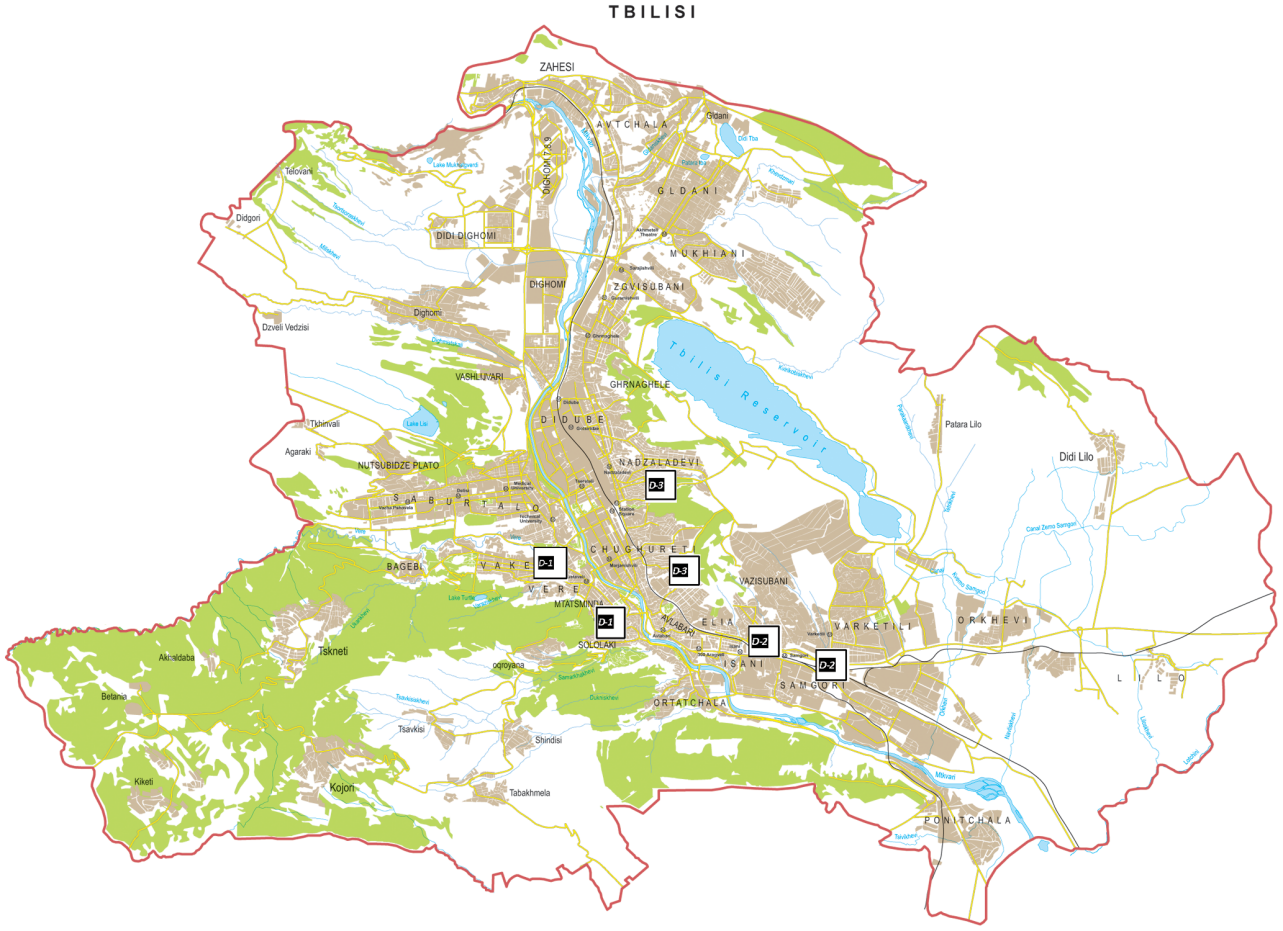
Map of Tbilisi with the three districts indicated. District 1 is located on the right bank of the Mtkvari River, whereas district 2 is located on the left, or Eastern, bank. District 3 is also described as ‘other Tbilisi, as it encompasses all parts of the city except Districts 1 and 2.

The situation is most urgent in the East Georgian city of Tbilisi, which has approximately 1.5 million inhabitants. Most of the natural VL foci are located in the very centre of the city, which are the oldest and most popular areas. Urban transmission is aided by the elongated shape of the city along the banks of the river Mtkvari (Kura), especially in areas located close to the hills and forests from where wild animals, including jackals and foxes, often appear. This situation facilitates the synanthropy between wild canids and stray and domestic dogs as these animals are known as reservoirs for *Leishmania* [8]. Since 1997 all VL cases have been reported from the left (Eastern) bank of the Mtkvari (see Figure 2).

In order to understand the spread of VL in Tbilisi and Kutaisi, three surveys were performed: (1) an infection screening of populations in selected districts of the two cities by Leishmanin Skin Test (LST); (2) a seroprevalence study of *Leishmania* infection in wild canines, stray and pets dogs; and (3) an entomologic identification of potential *Leishmania* vectors. We have identified alarmingly high prevalence rates in humans, vectors and dogs, especially in Tbilisi, although the emergence of VL in Western Georgia, where it has never been reported before, is almost equally serious.

## Materials and Methods

### Areas

Tbilisi is the capital and the largest city of Georgia (726 km^2^, 1,480,000 inhabitants), situated in the South Caucasus at 41°43′ North Latitude and 44°47′ East Longitude, lying on the banks of the Mtkvari (Kura) River. Highest elevation is 770 m and the lowest is 380 m above the sea level. Kutaisi is Georgia's second largest city, in the western region of Imereti and has approximately 186,000 inhabitants. It is located along both banks of the Rioni River, 221 km west of Tbilisi. The city lies at an elevation of 125–300 meters. For this study, Tbilisi was divided in three districts (see Figure 2). The climate of the Tbilisi area can be classified as humid subtropical, with relatively cold winters and hot summers, which constitutes a good seasonal habitat for sand flies.

### Ethics statement

Ethical clearance for conducting this study was secured by the Institutional Board of Review (IRB) of the National Center for Disease Control and Public Health (IRB00002150) in compliance with Georgian legislation and international bioethical frameworks. All volunteers were interviewed and written informed consent was obtained for participation in the study.

### Leishmanin skin test

Leishmanin was obtained by WHO from the Pasteur Institute, Teheran, Iran, made with phenolized *L. major* promastigotes [9]. The LST detects CL [10] as well as asymptomatic infection and cured VL although VL patients with active disease are generally LST negative as a result of a strong humoral response [11]. As such the LST is generally employed to assess prevalence of infection in a population rather than disease levels [12]. The sensitivity of the LST for cutaneous leishmaniasis was estimated as 88% in a recent study [13].

For the LST survey (during a 3 months period, June–August 2012) a cross-sectional study was carried out using a multi-stage cluster selection and probability proportional to size sampling [14], [15]. District #1 consists of the central areas located in the central part of city situated on the right (Western) side of the Mtkvari River, and is bordered by green parks, hills and forest. In this district VL cases have been registered every year since 1997; District #2 includes the areas located in the eastern part of city (left bank of the Mtkvari River), where VL cases have consistently been registered since 2000 (incidence lower than in District #1); District #3 consists of the remaining parts of Tbilisi where VL cases also have been permanently registered since 2000 (comparable less than in the first two districts) [official disease records, National Center for Disease Control (NCDC), Tbilisi, Georgia]. A total of 981 LST were performed within the thus-defined districts in Tbilisi (816), and in Kutaisi (163). After cleaning the skin over the flexor surface of the forearm with 70% alcohol, 0.1 ml of the antigen, containing standard amounts of *Leishmania* promastigotes, was administered intradermally employing single use insulin syringes. Skin test results (indurations) were read at 48 hours, although a small number was tracked for a 72 hours reading, according to the WHO recommendations [16]. Indurations were considered to be positive if they were at least 6 mm in diameter.

### Seroprevalence study of reservoirs

In June–August 2011/2012 blood-serum specimens from 1571 asymptomatic dogs were sampled in Tbilisi (623 pet and 670 stray dogs) and Kutaisi (151 pet and 50 stray dogs). The pet dog sampling was performed in the same areas where the LST screening was performed. Samples were taken from almost all pet dogs, with a few exceptions due to lack of consent from the owners. The blood from the stray dogs was collected thanks to the Municipal Service of Emergency and Urgent Situations of both Tbilisi and Kutaisi. Wild animals were captured alive and released after blood collection during a 2-month period (September–October 2012), with written permission from the Ministry of Environment of Georgia. Blood samples from 77 wild canines (38 foxes and 39 jackals) were taken. Collected canine blood samples were placed into Vacutainer vials for serum and stored at 4°C. For the detection of VL antibodies in canine serum Kalazar Detect rK39 Rapid tests (InBios International Inc, Seattle, USA) were performed according to the manufacturer's instructions [17], [18]. This test detects the circulating antibodies to recombinant K39 antigen of *L. donovani-infantum* complex and is highly specific (100%) and sensitive (97%) in diagnosing symptomatic and asymptomatic infections [19], [20]. The test procedure involved adding 20 µl of serum to the absorbent pad on the bottom of the test strip. Test strips were placed into a well of a sterile 96 well plate, to which 2–3 drops of buffer solution (provided with the test kit) were added; results were read within 10 minutes.

### Vector surveys and description of sandfly collection sites

There is a variety of housing styles within the study area, including apartment blocks of 9 floors or more; however, some 90% are modest private homes constructed of brick or stone. Most of these are walled compounds, with courtyards and gardens containing a variety of trees including fruit trees. Within these areas, pens for animals including dogs, chickens and rabbits are frequent which offers a diversity of blood meal sources, resting and breeding sites for sand fly species. Windows and doors are unscreened, providing easy access for sand flies to residents.

Vector surveys were implemented in Tbilisi (June–October, 2011) and Kutaisi (June–October, 2012). The total number of collected sand flies was 873 (656 in Tbilisi and 217 in Kutaisi); of these, 516 were female. Sandflies being phototrophic [21], [22] Seven CDC miniature light traps (John W. Hock Company, Gainesville, FL 32606, USA) were used to collect sand flies during three consecutive nights per month in each area [23]. Traps were placed outside houses, one per family compound, within fenced or protected habitats, especially near animal pens or in courtyards close to houses, between 7 pm and 7 am. Collected female sand flies were morphologically identified, dissected for detection of *Leishmania* parasites and scored according to various parameters: blood feed/unfed; gravid/non gravid [24].

### Examination of sand flies for infection with *Leishmania*

Live female sand flies were removed from traps and transferred to 10–20% soapy water solution to clean and immobilize them; afterwards they were rinsed in clean distilled water and soaked for about 10 minutes in 1% sodium hypochlorite solution to disinfect them. Sand flies were dissected in sterile conditions (sterile dissecting needles on a sterile microscope slide in a drop of sterile phosphate-buffered saline (PBS)) according to the method of Lawyer et al. [25]. Two terminal segments of the sand fly abdomen containing the spermathecae and the guts were separated from the whole body. Midguts were transferred to a fresh drop of sterile PBS on another clean slide for identification. Each gut was covered with sterile, glass cover slip.

In positive sand flies we observed a dense infection with many parasites attached to the microvillar lining of the midgut wall and to the cuticular intima of the stomodeal valve. Tightly packed Nectomonads and haptomonads were observed in the thoracic midgut, behind the stomodeal valve, which was forming a “plug” with a high proportion of easily motile metacyclic parasites that escaped into the sterile dissection buffer. They were distinguished by a small cell body with a long flagellum and fast movement. These motile cell forms were clearly visible and distinguishable from other elements under the 40× magnification.

### Data analysis

Data statistical analysis was performed using SPSS Statistics v20 software (IBM). We analyzed variants to compare differences between the groups and determined statistical significance, with *P* values less than 0.05.

## Results

### 1. Prevalence of Leishmania infection in the human populations of Tbilisi and Kutaisi as assessed by Leishmanin Skin Test

The results with the Leishmanin Skin Test (LST), summarised in Table 1, revealed a high prevalence of LST positives in age groups 5–9, 15–24 and 25–59 years in Tbilisi District #1 (22.2%, 37.5% and 19.5%, respectively). Prevalence for the same age groups was significantly lower in Kutaisi, at 0% (*P* = 0.0062), 3.2% (*P* = 0.0018) and 5.2% (*P* = 0.0017; all Χ^2^ test), respectively, and the overall difference in prevalence, 19.3% *versus* 7.3%, was also highly significant (*P* = 0.00016). Other notable results include high prevalence in Tbilisi District #2 for ages 10–14 (28.6%; n = 21); Tbilisi District #3 ages 1–4 (21.7%; n = 23); ages 60 and above in Kutaisi (17.24%) (Table 1). While overall prevalence was not significantly different between Tbilisi districts (*P*>0.05), a clear difference was observed in total prevalence in Tbilisi compared with Kutaisi (*P* = 0.0019, Χ^2^ test; Table 1). We further compared LST-positivity rates between male and female subjects in the same age groups (Supporting Information Table S1). Whereas prevalence in male subjects (15.2% overall) and female subjects (11.7%) was not significantly different, a significant difference was observed specifically in the 25–59 age group (16.2% males versus 7.8% females LST positive; *P = 0.011*); this may reflect a different behaviour pattern in working age subjects, with a larger percentage of men working outdoors in various occupations, in addition to males spending more social time outdoors; this is consistent with Georgian society, especially in an urban environment.

**Table 1:** Prevalence of VL as determined by Leishmania Skin Test (LST) in different age groups, arranged by district.

Age group	District #1 (Tbilisi)			District #2 (Tbilisi)			Kutaisi			Other Tbilisi			Tbilisi Totals			P value
	N	Positive	(%)	N	Positive	(%)	N	Positive	(%)	N	Positive	(%)	N	Positive	(%)	X^2^ test
**1–4**	9	0	0.0	51	4	7.8	35	5	14.3	23	5	21.7	83	9	10.8	NS
**5–9**	18	4	22.2	70	14	20.0	31	0	0	12	0	0	100	18	18	0.013^1^
**10–14**	20	3	15.0	21	6	28.6	44	4	9.1	18	3	16.7	59	12	20.3	NS
**15–24**	16	6	37.5	30	5	16.7	31	1	3.2	30	5	16.7	76	16	21.1	0.037^1^
**25–59**	77	15	19.5	157	15	9.6	116	6	5.2	61	6	9.8	295	36	12.2	0.034
**60 plus**	21	3	14.2	34	5	14.7	29	5	17.2	20	1	5.0	75	9	12.0	NS
**Total**	**161**	**31**	**19.3**	**363**	**49**	**13.5**	**286**	**21**	**7.3**	**164**	**20**	**12.2**	**688**	**100**	**14.5**	**0.0019**

P values in the final column are from comparisons between the overall prevalence in Tbilisi and in Kutaisi.

1 , using the Fischer Exact Probability test (two-tailed) because of low Expect values.

### 2. Assessment of infection in canines in and around Tbilisi by rK39 test

Based on the results of the rK39 test, we found that the highest proportion of seropositive pet dogs is present in District #2 (82/292 pet dogs, 28.1%) and District #1 (24/89; 27%) in Tbilisi; in District #3 the percentage was 21.5% (52/242). Surprisingly, the percentage was also quite high in Kutaisi, as 17.3% of pet dogs were positive (26/150), even though the first few cases of human VL in this city were reported only a few years ago. Table 2 shows a breakdown of rK39-positivity rates in pets, stray dogs and wild canids showing a highly significant (*P* = 5.3×10^−7^; 3-way X^2^ test) difference between those groups, with almost a quarter of all pet dogs testing positive as opposed to only 2.6% in wild canids. A further stratification was applied for the pet dogs, by age and breed (Table 2), and a X^2^ test confirmed that age and breed had a significant (*P*<0.001) influence on test outcome (prevalence). The table further shows that in particular the age of the dog is a main determinant, with increased age increasing infection rate, rather than the size of the breed.

**Table 2:** Results of the rK39 test for domestic (pet), stray and wild canines in and around Tbilisi, Georgia.

Breeds	Small			Medium			Large			Pets		
	ages ≤1.5	ages 1.5–5	age >5	ages ≤1.5	ages 1.5–5	age >5	ages ≤1.5	ages 1.5–5	age >5	TOTAL	Stray	Wild
Total	69	84	48	75	123	56	110	137	73	775	720	77
Positive	11	23	14	11	37	20	7	37	24	184	115	2
(%)	15.9	27.4	29.2	14.7	30.1	35.7	6.4	27.0	32.9	23.7	16.0	2.6

There was a significant association between age/breed and the percentage of dogs testing positive (P<0.001; X^2^ test).

### 3. Phlebotomus species in Tbilisi and Kutaisi as vectors for leishmaniasis

We identified five *Phlebotomus* species of three subgenera in Tbilisi and 3 of these species were also found in Kutaisi. Sand fly infectivity rates, species composition and the primary vectors have previously been reported to be different on either side of the Mtkvari River [6]. Amongst the 5 *Phlebotomus* species in Tbilisi the most abundant were *P. sergenti* (43% of total) and *P. kandelakii* (45%); whereas no infected specimens of *P. sergenti* were found, *P. kandelakii* displayed an infection rate of 5.5%, with all the positives originating in District # 1 -(Table 3). In District #1, situated on the right side of the Mtkvari River, the most abundant species was *P. kandelakii*, with 69.8%. In District #2, only two sand flies species were identified and *P. sergenti* was by far the most abundant (91.8%). In the rest of Tbilisi (Districts 3) *P. sergenti* was also the most abundant (84%) *Phlebotomus* species but no infected sand flies were found in this area. The most abundant sand fly species collected in Kutaisi city were *P. balcanicus* (53.5%) and *P. halepensis* (45.8% with infectivity rate of 1.3%) - the two least prevalent *Phlebotomus* species in Tbilisi. These results show a very different sand fly population in the two cities.

**Table 3:** *Phlebotomus* species identified in Tbilisi and Kutaisi.

Species	District #1 (Tbilisi)			District #2 (Tbilisi)			Kutaisi			Other Tbilisi		
	N	Positive	(%)	N	Positive	(%)	N	Positive	(%)	N	Positive	(%)
*P. (Adlerius) halepensis*	7	0	0.0	2	0	0.0	66	1	1.5	1	0	0.0
*P.(Adlerius) balcanicus*	7	0	0.0	3	1	33.3	86	1	1.2	3	0	0.0
*P.(Paraphlebotomus) sergenti*	28	0	0.0	94	0	0.0	2	0	0.0	34	0	0.0
*P. (Larroussius) wenyoni*	19	0	0.0	1	0	0.0	0	0	0.0	0	0	0.0
*P.(Larroussius) kandelakii*	157	9	5.7	0	0	0.0	0	0	0.0	6	0	0.0
Total	218	9	4.1	100	1	1.0	154	2	1.3	44	0	0.0

Each sand fly was microscopically examined for *Leishmania* parasites; both the number and percentage of *Leishmania*-positive flies are listed.

## Discussion

We report here the first detailed survey of leishmaniasis prevalence in humans and canids, and of *phlebotomine* sand fly populations, in the two main cities of Georgia. Historical records as well as more recent clinical records show that leishmaniasis is more prevalent in the Eastern parts of the country. Consistent with this trend, we find highly significant differences in infection rates between the Eastern city of Tbilisi and the Western city of Kutaisi. Overall prevalence of infection, as measured by the standard LST test, was double the rate in Tbilisi (P = 0.0019); this was significant for most age groups and where it did not reach significance, this was mostly due to smaller sample sizes in Kutaisi.

Whereas LST does not detect 100% of all infections, especially during clinically active disease, potentially producing false negatives; however despite its long use, this test has never been associated with significant numbers of false positives. We thus contend that the numbers reported here are more likely to be an underestimation than an overestimation of true leishmaniasis prevalence in Georgia.

The sand fly infection rate was quite high in Tbilisi and Kutaisi, 2.8% and 1.3%, respectively. The species of *Phlebotomus* were also different according to regions, with *P. sergenti* and *P. kandelakii* in Tbilisi and *P. halepensis* and *P. balcanicus* in Kutaisi. From this we surmise that the introduction of the disease to the Kutaisi region involved the adaptation of *L. infantum* to different vectors after the introduction of infected hosts to this region, with its distinct, endogenous sand fly population.

There is no historical record of sandfly infestation in the Kutaisi area, but the last sandfly survey of Western Georgia we are aware of dates from 1956 [26] and this study reported only a very low number of *P. chinensis*, and only in the Zestafoni district (approx 30 km South-east of Kutaisi). Apart from this, we have found no mention of leishmaniasis, or its vectors, in Kutaisi or the surrounding region in Western Georgia in the literature or in clinical records [8], [26]. Here, we report specimens of both *P. halepensis* (43% of total sand fly population in Kutaisi) and *P. balcanicus* (56% of total) infected with *Leishmania* parasites in Western Georgia (first time described in the country). Using the models of Pampiglione et al. for Mediterranean leishmaniasis [27], [28], the low seropositivity rate in the 15–24 and 25–59 age groups in Kutaisi, relative to age groups 1–4 and 10–14 (Table 1), suggests that this VL focus was developed relatively recently, as in old foci infection rates increase with age. Although we find the highest incidence in the 60-plus age group, this represents only 5 positives and we would hesitate to present this as evidence that Kutaisi is in fact an old focus. As the overall prevalence in Kutaisi is lower than in Tbilisi, the Kutaisi focus is also clearly less active but we need to emphasize that the results presented here constitute the first research on VL in Kutaisi since the first case of this disease was reported there, in 2007 [official statistical data of NCDC], and constitute a relatively small dataset.

The presence of infected *P. halepensis* in Western Georgia is potentially significant for a further reason: while this species is a suspected vector for *L. infantum* [29] it is also a suspected vector for *L. major*, and experimental infections with this species, at least, have been reported [30] While there is no proof of transmission of *L. major* in Georgia this possibility has never been investigated, and the presence of a potential vector in both eastern and western Georgia is of concern. Pratlong et al recently described the epidemiological features of Old World cutaneous leishmaniasis foci, based on the isoenzyme analysis of 1048 strains, and list a confirmed *L. major* strain [31] (as well as an *L. donovani* strain [32]) from Georgia. While it is not known whether this strain originated from local transmission or from a traveller infected elsewhere, 5 to 10 cases of cutaneous leishmaniasis are annually reported in Georgia [official disease records, National Center for Disease Control (NCDC), Tbilisi, Georgia]. However it is not currently known whether this is caused by *L. infantum*, which can also be responsible for cutaneous leishmaniasis [33], [34], or by *L. major* as suggested in the publication by Pratlong [31].

Within Tbilisi, the highest prevalence of LST positive subjects was found in central districts of the city situated on the right side of Mtkvari River (District 1), which has the highest population density in Tbilisi. Although this failed to reach statistical significance from the other two city areas sampled it correlates with the high number of seropositive pet dogs in that area and the highest diversity of *Phlebotomine* sand fly species in this district (Table 3).

The high LST prevalence among adult males compared to adult females in Tbilisi can be explained by their more frequent contacts with the vectors. The social pattern is that in summer time adult males spent a significant part of the evening with their neighbours and/or friends in either their own gardens or in nearby open spaces. At the same time most of adult females are doing housekeeping work and their social life is also far more indoors. A positive LST result is thought to indicate durable cell-mediated immunity after asymptomatic infection or clinical cure of VL and it persists in immunocompetent patients [35].

According to our observations during this study, for those dogs that were clinically suspected to have leishmaniasis, the rK39 test was weakly positive [19]. The seropositivity among pet dogs was found to be proportionate to their age as previously reported in other countries [18]. Seroprevalence was also high among stray dogs, however much less than in pet dogs (*P* = 0.00017), which means that the cycle is typically domestic. Indeed, most of the cases happen in houses with back gardens and chicken shelters, an appropriate environment for sand fly breeding, where dogs also cohabitate. A possible reason for the comparatively low prevalence in stray dogs may be the frequent movement of these animals within and outside the city, taking in areas of low population and/or vector density. Therefore, urgent control measures should focus on infected domestic dogs and vector control in the potential breeding sites around the house. We think that the roaming behaviour of stray dogs limits the contact with VL vectors but potentially contributes to the spread of the disease to new areas, developing new VL foci. The present study also reports for the first time that apparent VL being positive in the rK39 test was observed in wild foxes and jackals in Georgia. This appears to prove the hypothesis of Bardjadze, who suggested that these animals (especially the fox) are the reservoirs in non-permanent VL foci in this country [8]. However, it must equally be noted that we found only 2/77 wild canines (1 fox and 1 jackal) to be positive for rK39, a prevalence much below that of pet and stray dogs. This result seems consistent with the observations of Courtenay et al (2002) [36], who also observed low infection rates in wild foxes and concluded these are not important for the spread of *L. infantum* infection.

The transmission in active urban foci is thus from domestic dogs to human and, in the densely populated urban environment, appears to be much higher than in sylvatic environments. Thus, while we confirm that foxes and jackals do appear to carry leishmania parasites in non permanent foci of the Eastern part of the country they are not considered the main source of this disease in permanent foci such as Tbilisi and Kutaisi, in part due to their seasonal migration uplands in the summer, which is the disease transmission period.

We determined 5 *Phlebotomus* species in the selected districts of Tbilisi. The sand fly season starts in the last weeks of June or early July and ends in the middle of September [8]. The sand fly population peaks in the middle of July and starts to come down after the middle of August. The number of these vectors is strongly dependent on climate and environmental conditions [6], [7].The data summarised in Table 3 show that vector composition was different on either side of the Mtkvari River, even though the overall infection rate was very similar (*P*>0.05). In District 1, *P. kandelakii* was by far the most prevalent species (71.7%), whereas this species was not found in District 2, where *P. sergenti* was dominant (94.1%); *P. sergenti* was also the dominant sand fly species found in the rest of Tbilisi (77.3%). *P. kandelakii* was found to be infected with *Leishmania* parasites. No infected sand flies were found in District 3 (‘other Tbilisi’), but this could reflect the comparatively low number of flies sampled in this area.

This study shows that Tbilisi is an active focus for VL with very high infection prevalence in pets, in stray dogs and in humans as determined by the LST and rK39 tests. The infection rate in sand flies is also high, consistent with a recent report on the sand fly population in Tbilisi [37]. The microclimate of this city and the social behaviour of the population create conditions that are very favourable for the sand flies and for the spread of the infection. We demonstrate that almost the entire population of Tbilisi is at risk from VL, including all age groups, and in all districts, in large part because of the high percentage, and number, of seropositive dogs in the city. The outcome of the LST survey shows that a very significant percentage of the population has already been in contact with the parasite, although this does not imply that all will develop the clinical manifestations.

The comprehensive survey described in this manuscript has for the first time documented the very significant risk to the Georgian population from visceral leishmaniasis, and that its transmission is spreading to the west of the country. The results obtained will allow the Georgian health authorities to initiate control measures to reduce the high urban transmission rates responsible for the current outbreak, which displays the largest dimensions in many years, and to formulate a national strategy for leishmaniasis prevention and for improving treatment efforts to protect its population and economy from this very severe disease, of which the capital is especially at risk. In order to arrive at a comprehensive national strategy, however, it will be necessary to expand this survey to other districts of Georgia and to map the environmental risk factors not only in known areas of transmission but also at the national level to anticipate the progression of the disease to other vulnerable areas.

## Supporting Information

Table S1 Prevalence of *Leishmania* seropositives in various age groups, arranged by gender, as determined by the LST test.^1^, no statistical difference between Male and Female groups, by Χ^2^ test. ^2^, *P* = 0.011 by Χ^2^ test.
(DOCX)
