# Supplementary material for: Epidemiology of Visceral Leishmaniasis in Georgia
Source: PLoS Negl Trop Dis. 2014 Mar 6;8(3):e2725. doi: 10.1371/journal.pntd.0002725 (PMC3945224; doi:10.1371/journal.pntd.0002725)
Supplement: Table S1 — Prevalence of Leishmania seropositives in various age groups, arranged by gender, as determined by the LST test.1, no statistical difference between Male and Female groups, by Χ2 test. 2, P = 0.011 by Χ2 test. (DOCX) [file pntd.0002725.s001.docx]

**Supporting Information**

Table S1. Prevalence of *Leishmania* seropositives in various age groups, arranged by gender, as determined by the LST test. ^1^, no statistical difference between Male and Female groups, by Χ^2^ test. ^2^, *P*= 0.011 by Χ^2^ test.

| **Age group (years)** | **Male** | | | **Female** | | | **Total** | | |
| --- | --- | --- | --- | --- | --- | --- | --- | --- | --- |
|  | N | Positive | (%) | N | Positive | (%) | N | Positive | (%) |
| 1-4 | 65 | 6 | 9.2 | 53 | 8 | 15.1^1^ | 118 | 14 | 11.9 |
| 5-9 | 66 | 12 | 18.2 | 72 | 13 | 18.1^1^ | 138 | 25 | 18.1 |
| 10-14 | 50 | 7 | 14.0 | 53 | 9 | 17.0^1^ | 103 | 16 | 15.5 |
| 15-24 | 44 | 6 | 13.6 | 63 | 11 | 17.5^1^ | 107 | 17 | 15.9 |
| 25-59 | 117 | 19 | 16.2 | 294 | 23 | 7.8^2^ | 411 | 42 | 10.2 |
| 60 & above | 34 | 7 | 20.6 | 70 | 7 | 10.0^1^ | 104 | 14 | 12.5 |
| Total | 376 | 57 | 15.2 | 605 | 71 | 11.7^1^ | 981 | 128 | 13.5 |
